# Prediction of acute mountain sickness from overnight pulse oximetry during ascent to Everest Base Camp: a field-based machine learning study

**DOI:** 10.3389/fphys.2026.1939874

**Published:** 2026-09-30

**Authors:** Helen Abbott, Luke R. Cutts, Ben Stanley, Suniva Kansakar, Sophie E. Broughton, Taea Scott, Sam Davis, Sarah Clarke, Tim Clark, Ciaran Simpkins, Johnathan Goves, Ewein Howes, Kyle T. Pattinson, Adrian Garcia, William Trender, Arthur R. Bradwell, Santosh Baniya, Mark Edsell, Kimberly Ashdown, Kelsey E. Joyce

**Affiliations:** 1 Research Software Group, Advanced Research Computing, University of Birmingham, Birmingham, United Kingdom; 2 Salford Royal, National Health Service (NHS) Trust, Salford, United Kingdom; 3 Birmingham Medical Research Expeditionary Society, University of Birmingham, Birmingham, United Kingdom; 4 Anaesthetics Department, Lancashire Teaching Hospitals, Royal Preston Hospital, Preston, United Kingdom; 5 Queen Elizabeth II, University Hospitals National Health Service (NHS) Trust, Birmingham, United Kingdom; 6 Medical School, University of Birmingham, Birmingham, United Kingdom; 7 Occupational Performance Research Group, University of Chichester, Chichester, United Kingdom; 8 Anaesthesia & Intensive Care Medicine, Royal Blackburn Teaching Hospital, National Health Service (NHS) Trust, Salford, United Kingdom; 9 United Lincolnshire Hospital, National Health Service (NHS) Trust, Lincoln, United Kingdom; 10 Nuffield Department of Clinical Neurosciences, University of Oxford, Oxford, United Kingdom; 11 Medical School, University of Cardiff, Cardiff, United Kingdom; 12 H2 Cognitive Diagnostics, London, United Kingdom; 13 Emergency Medicine, Western Regional Hospital, Pokhara Academy of Health Sciences (PAHS), Pokhara, Nepal; 14 Anaesthesia and Critical Care, Royal Brompton Hospital, London, United Kingdom; 15 School of Sport Exercise and Rehabilitation Sciences, University of Birmingham, Birmingham, United Kingdom; 16 Human Integrative Physiology Lab, Mayo Clinic Arizona, Scottsdale, AZ, United States

**Keywords:** acute mountain sickness, high altitude, hypoxia, machine learning, pulse oximetry, risk prediction, wearable m5onitoring

## Abstract

**Background:**

Acute mountain sickness (AMS) affects a large proportion of individuals ascending to high altitude every year. While AMS is not life threatening, if left untreated it can progress to serious illness (e.g., high altitude cerebral oedema). Current assessment tools rely on subjective symptom reporting and do not enable prospective prediction. Objective, field-deployable methods for early risk stratification remain limited. This study evaluated whether physiological biomarkers derived from overnight pulse oximetry can predict subsequent AMS during ascent to Everest Base Camp (EBC).

**Methods:**

A field study was conducted in the Khumbu region of Nepal. Healthy lowlanders (n = 139–144 across datasets) underwent overnight pulse oximetry recordings at Namche Bazaar (3,440 m) and Dingboche-level (4,371-4,730 m). A total of 125 physiological features were extracted from continuous, overnight oxygen saturation (SpO_2_) and heart rate recordings. Supervised machine-learning models were trained to classify physician-diagnosed AMS at Lobuche (~4,940 m) using: 1) physiological biomarkers alone and 2) biomarkers combined with clinical and contextual metadata. Model performances were assessed using repeated stratified k-fold cross-validation, with area under the receiver operating characteristic curve (AUC) as the primary metric.

**Results:**

Biomarker-only models demonstrated consistent above-chance discrimination at both altitudes (mean AUC 0.74 at both Namche and Dingboche-level). At lower altitude, predictive performance was achieved using a small set of physiological features, and inclusion of metadata did not improve model performance. At higher altitude, combining biomarkers with contextual variables improved discrimination (AUC 0.84 ± 0.08; sensitivity 0.73 ± 0.23). Selected features were primarily related to nocturnal oxygenation burden, desaturation dynamics, and heart rate variability.

**Conclusion:**

Overnight pulse oximetry captures physiological signals associated with subsequent AMS development prior to symptom onset. These findings support the feasibility of objective, non-invasive risk stratification during early ascent. Integration of wearable monitoring with computational modelling may provide a complementary tool to support preventative decision-making along high-altitude trekking routes.

## Introduction

Acute mountain sickness (AMS) is a syndrome that affects a large proportion of individuals ascending to high altitude (>2,500 m) every year (Karinen et al., 2008; Croughs et al., 2014). While AMS is not life threatening, if left untreated it can progress to severe AMS or more serious and life-threatening altitude illnesses such as high-altitude cerebral or pulmonary oedema (HACE or HAPE, respectively), which require immediate medical intervention and often evacuation. Despite being less severe than HACE and HAPE, AMS is the most prevalent of the three main altitude-related illnesses. More specifically, AMS has been observed to have an incidence rate of ~30% in the Khumbu region along the Everest Base Camp (EBC) route (Basnyat et al., 1999), one of the busiest trekking routes globally. Annual records indicate that more than 55,000 individuals, including trekkers, porters, and guides, ascend through this region each year (Conservation, 2021). Thus, the prevention of AMS and/or prediction prior to the onset of symptoms is of primary importance.

AMS prevention can, in part, be aided by adopting a conservative rate of ascent that does not exceed an increase in sleeping altitude by 500 m per day when above 3,000 m (Luks et al., 2024). Unfortunately, this is not always possible due to fixed schedules of commercial trekking companies. AMS prophylaxis may also involve pharmacological intervention such as acetazolamide (Joyce et al., 2018). Despite its effectiveness, acetazolamide is known to have unwanted side effects (including paraesthesia, polyuria diuresis, fatigue, and impaired exercise capacity) that can cause detrimental task-specific implications for climbers (Bradwell et al., 2018; Schmickl et al., 2020; Hand and Joyce, 2025). This can discourage its use and, therefore, increase the risk of AMS. Similarly, many individuals may be unnecessarily prescribed acetazolamide prophylactically, as predicting AMS before symptom onset remains challenging, particularly when symptoms are sub-clinical or resemble other travel-related conditions. Whilst AMS can be tracked with subjective symptom scoring such as the Lake Louise Score Criteria (Roach et al., 2018) or the Environmental Symptoms Questionnaire (Sampson and Kobrick, 1980), these questionnaires are not predictive, only tracking symptoms as they develop. Therefore, there is a need for a quick, field-deployable method to prospectively predict AMS, irrespective of acetazolamide use.

Recent evidence suggests that overnight pulse oximetry may help identify individuals at increased risk of AMS. A recent systematic review concluded that lower oxygen saturation during ascent is associated with subsequent AMS development but highlighted substantial methodological heterogeneity (Goves et al., 2024). Subsequent work demonstrated relationships between overnight pulse oximetry metrics and AMS susceptibility, while our previous study extended these findings by developing predictive classification models; however, both studies were limited by small sample sizes (n = 18 and n = 20, respectively) (Joyce et al., 2024; Cutts et al., 2026). Consequently, the generalisability of overnight pulse oximetry-based AMS prediction in larger real-world trekking populations remains unclear.

The present study aimed to address this limitation through substantially increasing the sample size compared with previous model-development studies. The primary aim was to develop predictive models of AMS risk using: (1) physiological biomarkers derived from overnight pulse oximetry alone; and (2) these biomarkers in combination with clinical and contextual factors. The intended use of the model is as a screening and risk stratification tool to identify individuals at increased risk of developing AMS during ascent, to support decisions regarding monitoring, pacing, and preventative strategies.

## Methods

### Ethical approval

This study was approved by the University of Chichester ethics committee (Ethical Review No: 2324_79) and the Nepal Health Research Council (NHRC number: 555_2024). This study was carried out in accordance with the Declaration of Helsinki 2024 except for database registration. Methodology was conducted/reported in accordance with the STAR reporting guidelines, which are specific to altitude research (Brodmann Maeder et al., 2018), and TRIPOD-AI reporting recommendations for prediction models where applicable (Collins et al., 2024).

### Eligibility, recruitment, and consent

Healthy lowlanders (resident < 2,500 m) between the age 18–65 years were considered for inclusion. Individuals with significant cardiac, metabolic, neurologic, or respiratory disease were excluded. For example, individuals with chronic obstructive pulmonary disease were excluded as they are known to have a higher incidence of sleep disordered breathing, a factor that would negatively impact our analysis (Sánchez-Mascuñano et al., 2017). Individuals with any history of atrial fibrillation (currently controlled or uncontrolled) were excluded. Individuals with a history of smoking (>20 pack years) or recently quit smoking (within last 6 months) were excluded, as some studies have suggested a possible protection from altitude sickness with smoking (Sánchez-Mascuñano et al., 2017). Individuals taking any medication prophylactically for mountain sickness were still eligible for this study.

Recruitment and enrolment took place in Namche Bazaar (3,440 m). Participants were recruited from the general population of trekkers already planning to ascend to EBC. All participants underwent medical assessment at enrolment at Namche Bazaar.

Participants were scheduled to have additional overnight stops at Dingboche (4,410 m) and Lobuche (4,940 m) (**Figure 1**). For the purposes of this study, Dingboche included equivalent acclimatisation locations at similar altitude (Pheriche, 4,371 m; *n* = 14, and Chhukung, 4,730 m; *n* = 3), hereafter referred to as “Dingboche-level”. These locations were grouped due to their comparable altitude range and equivalent role in the ascent profile.

**Figure 1:**
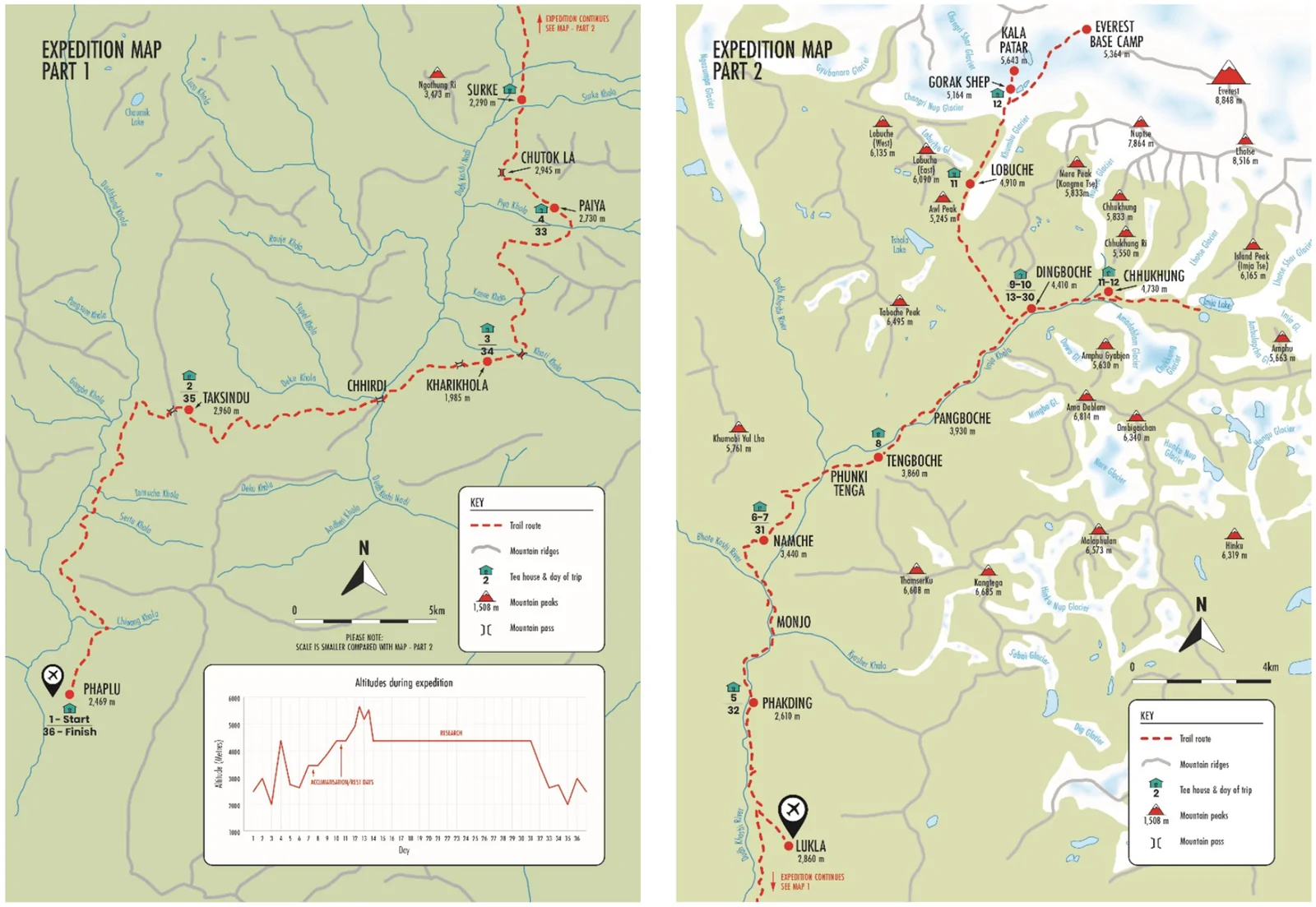
Study area and expedition route in the Khumbu region of Nepal. Combined maps showing the Everest Base Camp trekking route and the locations at which participant recruitment, physiological measurements, and outcome assessments were conducted. Major study locations included Namche Bazaar, Dingboche-level acclimatisation sites, Lobuche, and Everest Base Camp. Reproduced with permission from Arthur R Bradwell.

Participants were provided with information sheets about the study with adequate time to ask questions prior to consent. Written informed consent was obtained from all participants in the field prior to participation. Participants were permitted to withdraw at any time without the need to provide any explanation.

### Study design

Participants had three in-person contacts with the research team: Namche, Dingboche-level, and Lobuche (**Figure 2**). As part of enrolment in Namche, participants were asked to complete a general health history questionnaire, which included history of altitude illness (AMS, HAPE, HACE, and high-altitude headache). This was followed by a brief routine medical examination conducted by the research expedition Medical Officer (as described in next section), and the Fitzpatrick Skin Tone assessment (Fors et al., 2020). Oximetry recordings were completed on the participants’ second nights in Namche and Dingboche-level as per **Figure 2**. Finally, medical examinations were repeated in Lobuche.

**Figure 2:**
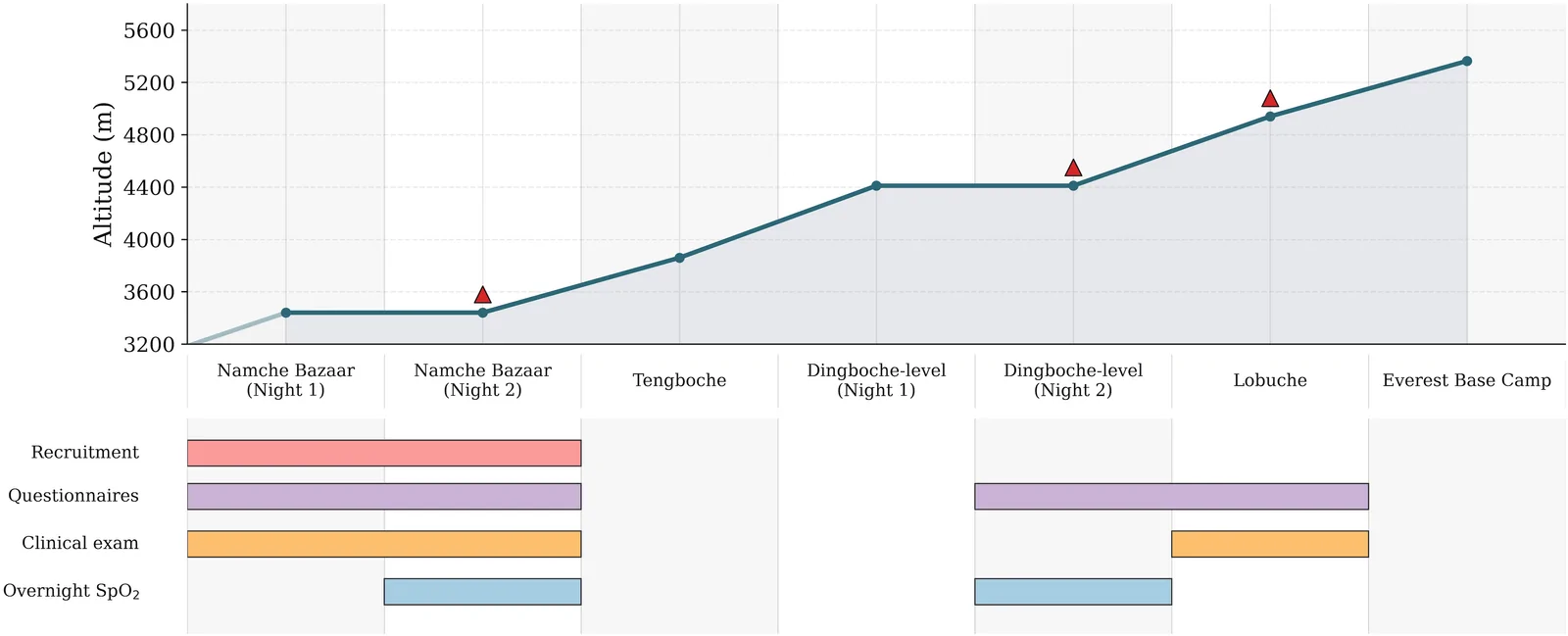
Study design and high-altitude ascent profile in the Everest region. Altitude profile across the expedition route from Namche Bazaar to Everest Base Camp (EBC), showing overnight stops and acclimatisation schedule. Triangles indicate locations of in-person research contact (Namche, Dingboche-level and Lobuche). Dingboche-level includes participants from Dingboche (4,410 m), Pheriche (4,371 m), Chhukung (4,730 m). The lower panel summarises the study protocol, including participant recruitment, questionnaire completion, continuous physiological monitoring, clinical examinations, and overnight oxygen saturation (SpO_2_) recordings. Questionnaires included the Lake Louise Scoring Criteria, Groningen Sleep Quality Scale (GSQS), and altitude sickness history; history was collected at Namche only. Shaded regions distinguish consecutive days/locations.

An overview of the study workflow and analytical pipeline is shown in **Figure 3** (data collection procedures are described in detail in **Figure 2**).

**Figure 3:**
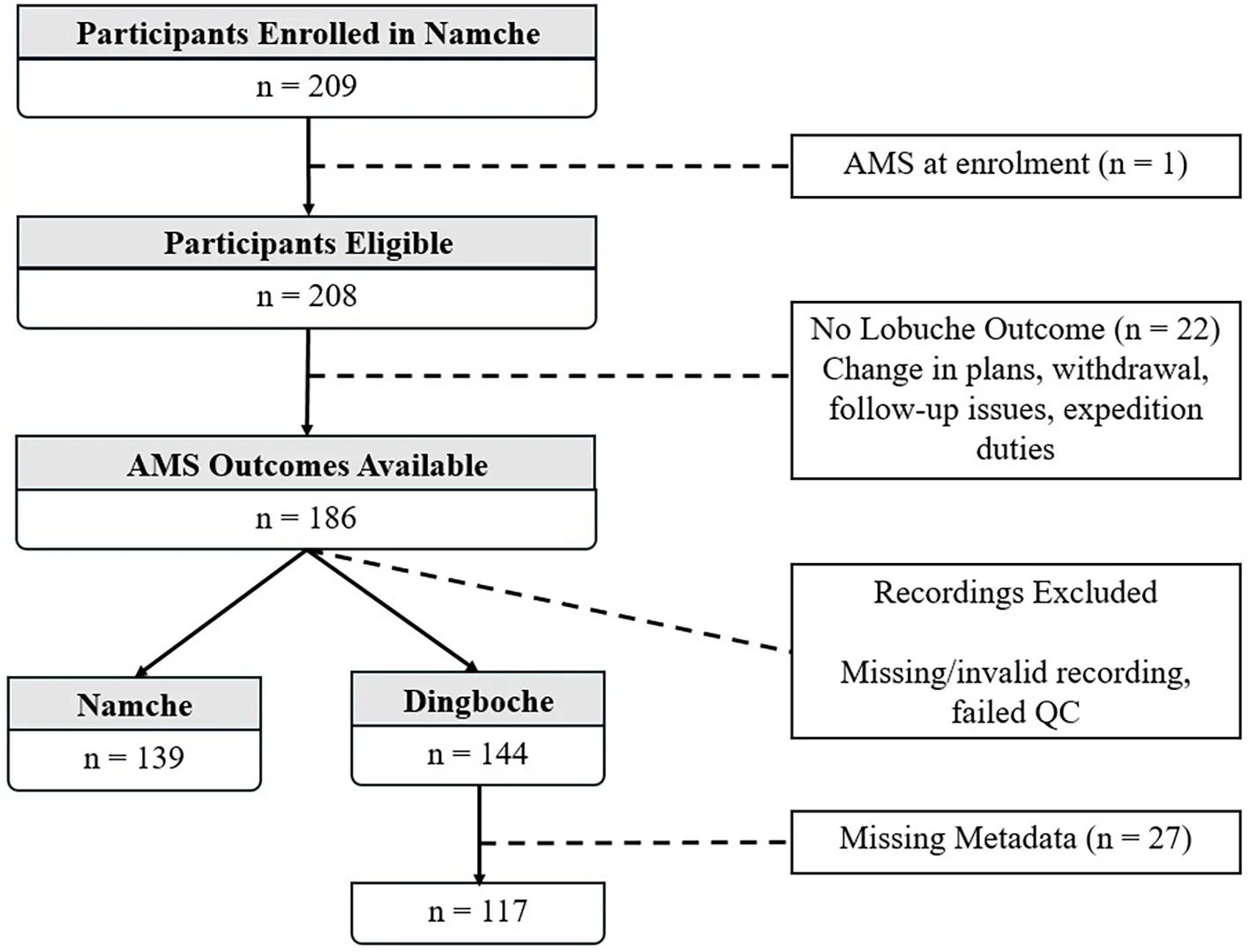
Participant flow and dataset derivation for machine-learning analyses. Flow diagram summarising participant recruitment, baseline exclusions, follow-up to Lobuche, outcome ascertainment, oximetry quality-control (QC) procedures, and construction of the four analytical datasets. Reasons for exclusion at each stage are shown. Final sample sizes differ between datasets owing to missing metadata and recording-quality requirements.

### Medical examinations

Medical examinations were conducted in Namche and Lobuche by a fully registered General Medical Council (GMC) physician to obtain a binary diagnosis of AMS. Medical exams encompassed a structured history and clinical exam, as well as LLS score. This composite assessment was carried out to provide a robust clinical diagnosis and reduce misclassification associated with reliance on LLS alone (Meier et al., 2017). If a participant was assessed to be positive for AMS at Namche, they were excluded from the study.

During medical examinations participants were also asked questions about their recent medication history (over the counter or prescribed), acetazolamide use, and alcohol consumption. Additionally, during medical examinations, details regarding participants’ ascent history and acclimatisation walks were recorded. Participants reported the sleeping altitude of each overnight stay and the date of arrival in Kathmandu. An average rate of ascent ( $m\;day^{-1}$) was later calculated by dividing the altitude gain from Kathmandu to the current assessment location by the number of days elapsed since arrival in Kathmandu. This value was used as a contextual variable in the subsequent metadata feature set.

Clinicians assessing AMS were not informed of oximetry-derived features used for model development.

### Outcome definition

The primary outcome was a binary diagnosis of AMS at Lobuche (~4,940 m). All predictor variables, including physiological biomarkers, symptom scores, and contextual metadata, were collected at earlier timepoints (Namche or Dingboche-level), prior to outcome assessment at Lobuche. This temporal separation minimised the risk of information leakage by ensuring that no outcome-defining variables were included as predictors.

### Questionnaires

Participants were asked to complete questionnaires the morning after oximetry recordings and in Lobuche (**Figure 2**). Questionnaires completed included: Lake Louise Score (LLS) (Roach et al., 2018), Groningen Sleep Quality Questionnaire (Jafarian et al., 2008), and recent medications history and alcohol use. The LLS was used to assess symptoms associated with AMS, including headache, gastrointestinal symptoms, fatigue, dizziness, and sleep disturbance (Roach et al., 2018). The GSQS assesses subjective sleep quality and consists of items relating to sleep disturbance and perceived sleep adequacy (Jafarian et al., 2008).

### Overnight oximetry procedures and recordings

Continuous overnight pulse oximetry was performed during the second night in Namche and Dingboche-level to maximise capture of acclimatisation effects.

If participants were only staying one night in these locations, the recording was collected anyway, with this factored into the ascent profile metadata. Overnight recordings for peripheral oxygenation (SpO_2_) and heart rate (HR) were collected using wrist worn oximeters (WristOx2 Model 3150, Nonin Medical) with accompanying flexible finger sensor with PureLight^®^ technology (Adult 8000J, Nonin Medical). Pulse oximeter application was demonstrated by researchers to participants prior to overnight recordings, with manufacturer video demonstration also provided. Participants were instructed to apply the oximeter immediately before bed and to unplug the sensor immediately upon waking. Participants were asked to apply the finger sensor to the ring finger of their nondominant hand.

#### Oximetry pre-processing and feature extraction

Second-by-second (1 Hz) raw recordings were offloaded from oximeters after every on-mountain recording. Data were processed in MATLAB (2024b, Mathworks, Natick, MA, USA), prior to feature extraction in Python (v3.12). Artefacts were identified using a delta filter (SpO_2_ < 50%, SpO_2_ >100% or changes > 4% in SpO_2_ or ≥ 6 bpm in HR between consecutive samples) (Taha et al., 1997; Levy et al., 2021b). A median filter (length 9) was applied to remove outliers and sharp changes (Levy et al., 2021a). Detected artefacts were removed from both signals and artefact-index (AI; percentage of the total raw overnight recording identified as artefact) was calculated for quality control. Finally, small gaps (≤3 samples) were filled using linear interpolation (Buekers et al., 2019).

A total of 125 biomarkers (see **Supplementary Material, Supplementary Tables 1–9**) were extracted using a modified version of PhysioZoo (Behar et al., 2023) adapted to accommodate file formats and integrate HR feature extraction.

### Data inclusion and missing data handling

Participants were included in each dataset if they had ≥4 hours of valid overnight oximetry recordings and corresponding outcome data. Recordings that failed to meet quality control criteria, based on artefact index thresholds or substantial non-physiological signal that could not be corrected during preprocessing, were excluded.

Missing data arose primarily from two sources: incomplete outcome assessment and oximetry recordings failing quality control. Of the 209 participants enrolled, 186 had a valid AMS outcome at Lobuche and were eligible for analysis. Missingness was primarily attributable to loss to follow-up, changes in trekking itinerary, logistical constraints, incomplete data capture, or recordings of insufficient quality. A subset of recordings was excluded due to anomalous or poor-quality oximetry data identified during preprocessing. One individual meeting physician-determined diagnostic criteria for AMS was excluded prior to participation. No further exclusions were made based on AMS symptoms at enrolment.

A complete-case analysis approach was used for all model development, whereby participants were included only if they had valid physiological recordings and outcome data. Missing metadata further contributed to reduced sample sizes in combined models. Imputation was not performed due to the modest sample size and the high dimensionality of the feature set. A participant flow diagram detailing recruitment, exclusions, loss to follow-up, quality-control exclusions, and derivation of the four analytical datasets is presented in **Figure 3**.

### Feature filtering for machine learning

All processed biomarkers, along with metadata from medical examinations and questionnaires were included as candidate features (**Supplementary Tables 1–10**).

Feature filtering was performed using Pearson correlation and domain-specific criteria. For highly correlated feature pairs (|r| > 0.95), the feature with the higher mean absolute correlation with other variables was removed. Variables overlapping with the outcome definition were excluded to prevent information leakage. Specifically, aggregated LLS scores were excluded, while individual symptom components from earlier timepoints were retained where appropriate.

### Machine learning techniques

Five supervised classification algorithms were evaluated: logistic regression, linear support vector machines (SVM), random forests, decision trees, and gradient boosting classifiers. These models were selected to represent a range of linear (logistic regression and linear SVM), tree-based (decision trees), and ensemble (random forest and gradient boosting) approaches.

Model performance was estimated using repeated stratified k-fold cross-validation (5 folds × 10 repeats). Within each outer cross-validation split, the dataset was partitioned into training and validation folds, with performance evaluated exclusively on the held-out validation data. For logistic regression and SVM, features were standardised using z-score normalisation, with scaling parameters estimated from training data only. Tree-based models were trained using unscaled features.

Feature selection was performed using Recursive Feature Elimination with Cross Validation (RFECV), implemented entirely within the training data of each outer split. RFECV recursively removed least informative features and selected the optimal subset using cross-validated performance. No validation data were used during feature selection or model fitting. Feature selection frequency across folds and repeats was recorded and reported, and the top 10 most frequently selected features were retained (**Supplementary Tables 10–14**). Final models were subsequently developed by iteratively retraining and evaluating combinations of these top-ranked features, with the optimal feature subset selected according to cross-validated predictive performance rather than selection frequency alone. To account for class imbalance in AMS prevalence, class weighting using *`class_weight=‘balanced’* was applied during model training, ensuring that minority class (AMS positive) observations contributed proportionally to model training.

Performance was assessed using accuracy, area under the receiver operating characteristic curve (AUC), F1 score, precision and sensitivity (recall). AUC was selected as the primary metric due to reduced sensitivity to class imbalance. To quantify uncertainty in performance estimates, 95% confidence intervals were derived using participant-level bootstrap resampling of aggregated out-of-fold predictions obtained during repeated nested cross-validation. Classification metrics are reported at a default probability threshold of 0.5, with additional threshold sensitivity analyses performed on pooled out-of-fold predictions (**Supplementary Material, Supplementary Tables 21–23**). Model calibration was assessed using calibration curves and the Brier score, calibration intercept, and calibration slope, calculated from pooled out-of-fold predictions across cross-validation splits.

The overall study and machine learning pipeline, including data preprocessing, biomarker extraction, feature filtering, and model development, is illustrated in **Figure 4**.

**Figure 4:**
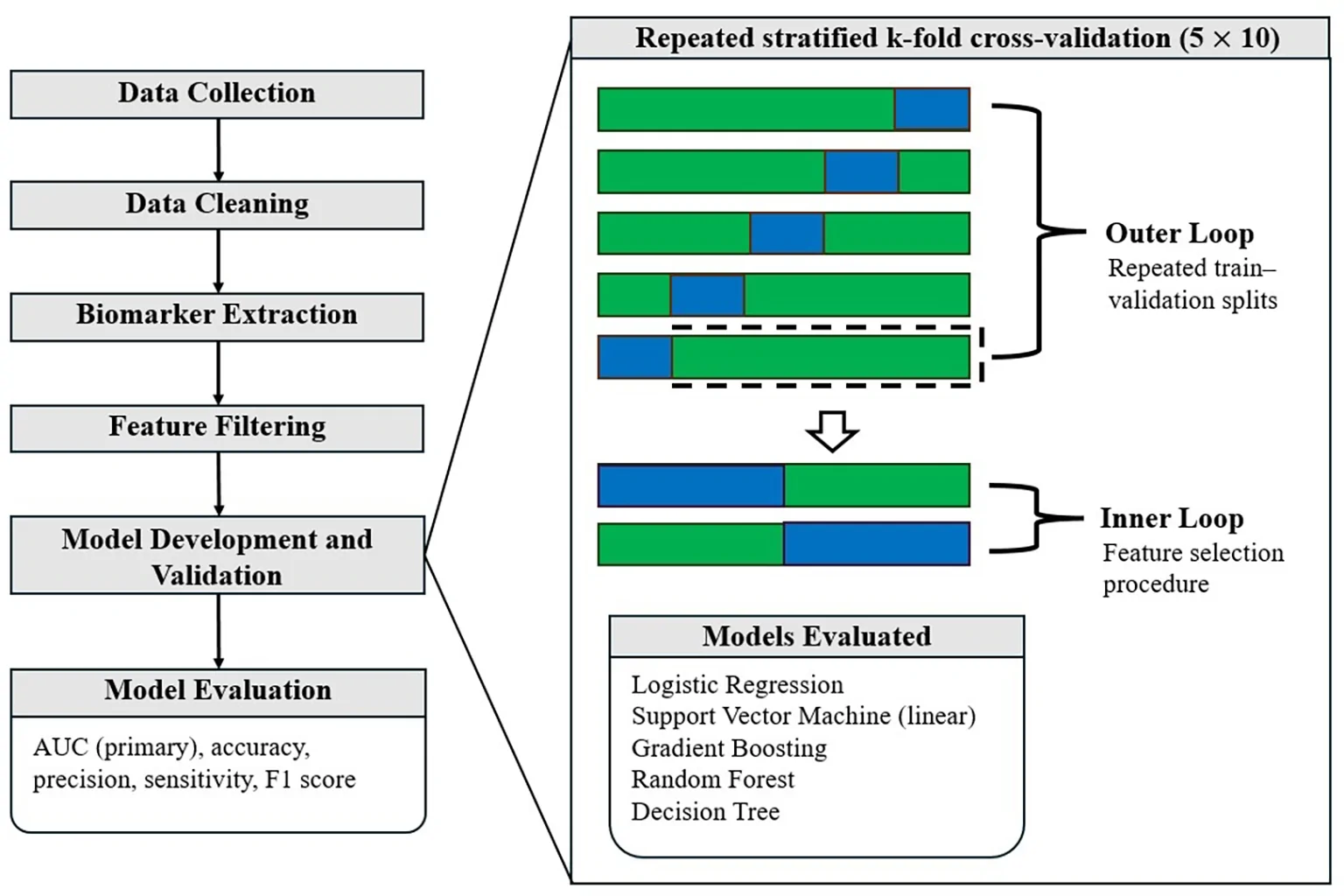
Machine learning pipeline for Acute Mountain Sickness (AMS) prediction. The pipeline involves stages of data collection (see **Figure 2**), signal preprocessing (cleaning and artefact removal), biomarker extraction, and feature filtering. Model development was performed using repeated stratified k-fold cross-validation (5 × 10), with nested feature selection (RFECV) conducted exclusively within training folds (inner loop). Multiple classifiers were evaluated, and performance was assessed on held-out validation data using area under the receiver operating characteristic curve (AUC) as the primary metric, with additional metrics including accuracy, precision, sensitivity, and F1 score.

Logistic regression demonstrated the most consistent performance and was selected as the final model for subsequent analyses. This study performs internal validation using repeated cross-validation; no independent external validation dataset was available. Model interpretability was assessed using SHapley Additive exPlanations (SHAP), with full SHAP analyses provided in the **Supplementary Material, Supplementary Figures 3, 4**.

## Results

Four datasets were analysed, defined by measurement location (Namche or Dingboche-level) and feature set (physiological biomarkers alone or combined with metadata). The binary outcome was physician-determined AMS at Lobuche (see Methods), used as the classification target for all machine learning models. Dataset characteristics are summarised in **Table 1**. AMS prevalence ranged from 17–22%, indicating moderate class imbalance. Sample size differences reflect exclusions due to missing data and quality control criteria. Baseline demographic characteristics for participants with available demographic and metadata information are summarised in **Table 2**.

**Table 1:** Characteristics of the four datasets used in this study.

Location	Altitude (m)	Features	Total participants	Participants with AMS diagnosed in Lobuche
Namche	3,440	Biomarkers	139	30
Namche	3,440	Biomarkers + Metadata	139	30
Dingboche-level*	4,371-4,730	Biomarkers	144	29
Dingboche-level*	4,371-4,730	Biomarkers + Metadata	117	20

*Dingboche-level includes participants from Dingboche, Chhukung or Pheriche. Acute mountain sickness, AMS. The table reports data collection sites, feature types, sample sizes, and the prevalence of the binary outcome (AMS diagnosed in Lobuche).

**Table 2:** Baseline participant characteristics stratified by Acute mountain sickness (AMS) outcome (participants with available demographic and metadata data).

Characteristic	Namche No AMS (n = 109)	Namche AMS (n = 30)	Dingboche-level* No AMS (n = 97)	Dingboche-level* AMS (n = 20)
Age, years	37.4 ± 12.7	37.8 ± 12.6	36.9 ± 12.8	34.8 ± 9.8
Male sex, n (%)	53 (48.6)	13 (43.3)	43 (44.3)	8 (40.0)

*Dingboche-level includes participants from Dingboche, Chhukung or Pheriche. Acute mountain sickness, AMS. Participant counts correspond to individuals with available demographic and metadata information. Sample sizes therefore reflect the biomarker + metadata datasets rather than the larger biomarker-only datasets. Acute mountain sickness, AMS.

Across all four datasets, logistic regression demonstrated the most consistent cross-validated performance and was selected for subsequent analyses. Results from other classifiers are not shown but are available in the data repository (10.5281/zenodo.20428956).

Using physiological biomarkers derived solely from overnight pulse oximetry data, models achieved above-chance discrimination at both study locations (**Figure 5**). Biomarker-only models demonstrated comparable discriminative performance in Namche and Dingboche-level, with mean cross-validated AUCs of 0.74 ± 0.09 (95% CI 0.64-0.83) and 0.74 ± 0.10 (95% CI 0.63-0.84), respectively (**Figure 5**; **Table 3**).

**Figure 5:**
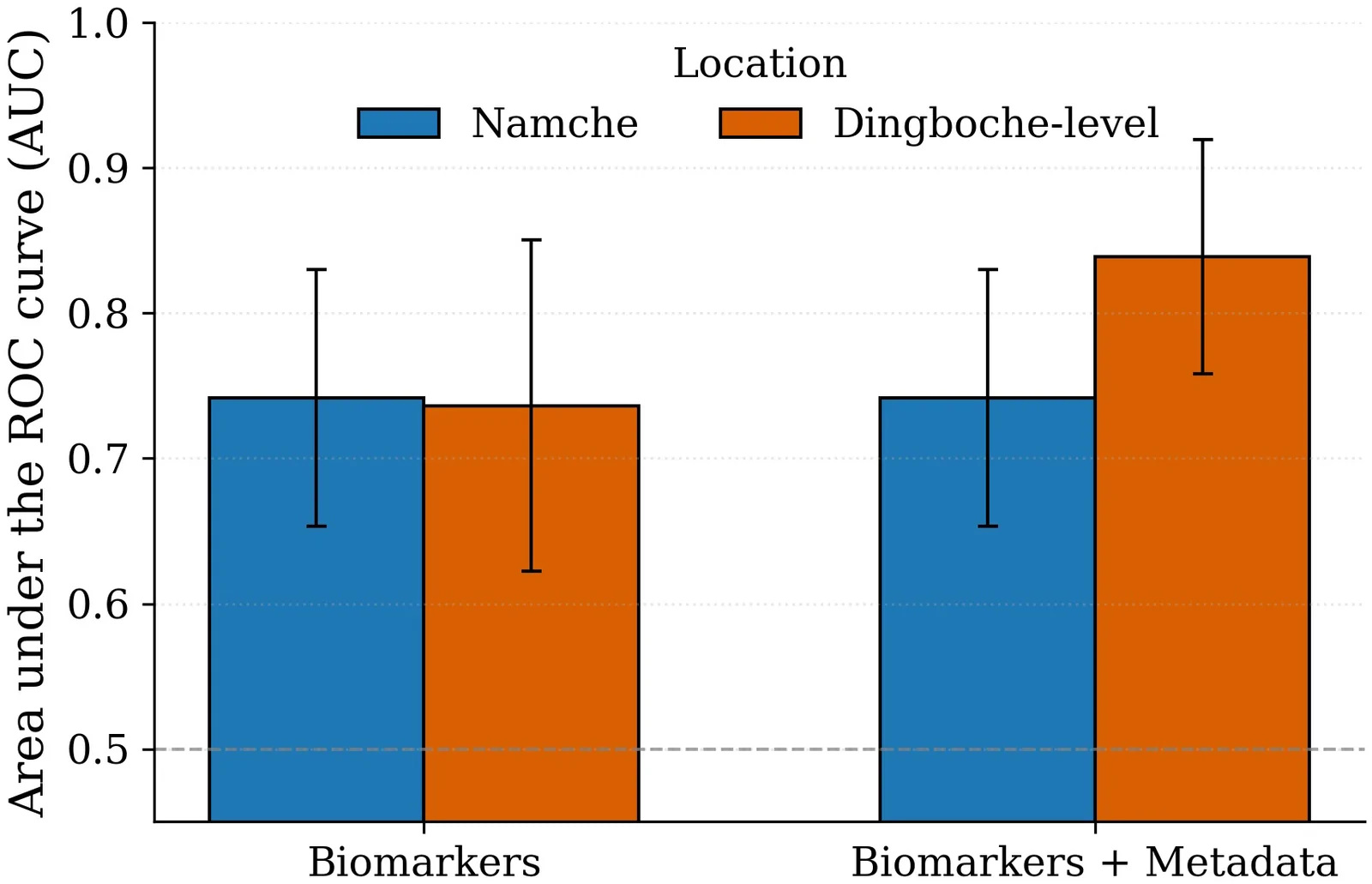
Cross-validated performance of logistic regression models for acute mountain sickness (AMS) prediction using overnight pulse-oximetry–derived physiological biomarkers, with and without additional demographic and clinical metadata, at Namche and Dingboche-level. Bars show mean area under the receiver operating characteristic curve (AUC) across repeated stratified cross-validation splits, with error bars indicating Standard Deviation. The dashed horizontal line indicates chance-level discrimination (AUC = 0.5). Dingboche-level includes participants from Dingboche, Chhukung and Pheriche.

**Table 3:** Cross-validated predictive performance of logistic regression models for acute mountain sickness (AMS) prediction using overnight pulse-oximetry–derived physiological biomarkers, with and without additional demographic and clinical metadata, at Namche and Dingboche-level.

Location	Feature Set	Accuracy	Precision	Sensitivity	F1
Namche	Biomarkers	0.68 ± 0.08 (0.58-0.74)	0.38 ± 0.10 (0.23-0.48)	0.69 ± 0.18 (0.49-0.83)	0.48 ± 0.11 (0.32-0.59)
	Biomarkers + Metadata	0.68 ± 0.08 (0.58-0.74)	0.38 ± 0.10 (0.23-0.48)	0.69 ± 0.18 (0.49-0.83)	0.48 ± 0.11 (0.32-0.59)
Dingboche-level*	Biomarkers	0.72 ± 0.08 (0.65-0.81)	0.39 ± 0.12 (0.25-0.53)	0.62 ± 0.2 (0.44-0.89)	0.46 ± 0.12 (0.33-0.61)
	Biomarkers + Metadata	0.77 ± 0.09 (0.69-0.85)	0.43 ± 0.14 (0.24-0.57)	0.73 ± 0.23 (0.47-0.91)	0.52 ± 0.15 (0.33-0.67)

*Dingboche-level includes participants from Dingboche, Chhukung and Pheriche. Values are reported as mean ± SD across repeated nested cross-validation runs. Bootstrap-derived 95% confidence intervals from participant-level aggregated out-of-fold predictions are shown in parentheses.

The inclusion of metadata had a location-dependent effect. In Namche, adding metadata did not improve performance, relative to the biomarker-only model. In contrast, at Dingboche-level, the inclusion of metadata was associated with higher values for all performance metrics, including AUC (0.84 ± 0.08; 95% CI 0.76-0.91), accuracy, precision, sensitivity, and F1 score (**Figure 5**; **Table 3**).

Discriminative performance across probability thresholds is shown in **Supplementary Material, Supplementary Figure 2**. Consistent with the AUC results, the Dingboche-level biomarker + metadata model demonstrated the strongest overall performance in both ROC and precision-recall space.

Classification metrics were calculated at a default probability threshold of 0.5. At this threshold, sensitivity exceeded precision across datasets, indicating that the models preferentially identified true AMS cases at the expense of increased false positives, consistent with a screening-oriented approach.

Threshold sensitivity analysis demonstrated the expected sensitivity-specificity trade-off. The Dingboche-level biomarker + metadata model achieved higher specificity at equivalent sensitivity than the biomarker-only models (**Supplementary Material, Supplementary Tables 21–23**). At lower thresholds (0.2-0.3), sensitivity exceeded 0.9; however, precision remained modest across all thresholds (≤0.42), indicating a substantial number of false-positives and reinforcing the model’s intended use as a screening rather than diagnostic tool.

Full model outputs are available in the data repository (10.5281/zenodo.20428956). Calibration analysis demonstrated moderate calibration, with Brier scores ranging from 0.157-0.208. All models showed some tendency to overestimate risk (**Supplementary Material, Supplementary Table 24; Supplementary Figure 5**).

Feature selection identified compact, location-specific predictor sets (**Table 4**). At Namche, model performance was achieved using four physiological features, and inclusion of metadata did not change the selected set. At Dingboche-level, nine features were retained, with the inclusion of metadata introducing two contextual variables (LLS headache and history of high-altitude headache). To evaluate the potential influence of the LLS headache component on model performance, further analysis was conducted excluding headache from the metadata feature set. Model discrimination remained largely preserved (AUC 0.81 vs. 0.84 in the primary model), suggesting that predictive performance was not primarily driven by headache-related information (**Supplementary Material, Supplementary Table 15**).

**Table 4:** Features selected for the best performing logistic regression models by location and feature set (biomarker-only vs biomarker + metadata).

Location	Feature set	Selected features
Namche	Biomarkers	DA_max sd_, CT_91_, F2S, SpO_2 Min_
	Biomarkers + Metadata	CT_91_, DA_max sd_, F2S, SpO_2 Min_
Dingboche-level*	Biomarkers	DS_sd_, HR_ZC_, PSD_peak frequency_, PSD_band kurtosis_, CA, DL_sd_, F2S, DD_100_sd_, SpO_2 SK_
	Biomarkers + Metadata	PSD_band kurtosis_, DL_sd_, HAH History, PRSAsa, F2S, HR_ZC_, LLS_Headache_, SpO_2 SK_, SpO_2 K_

*Dingboche-level includes participants from Dingboche, Chhukung and Pheriche.

Selected features spanned multiple physiological domains, as defined below:

Hypoxia burden

- CT_91_: Proportion of total recording time spent with SpO_2_ < 91%
- CA: Cumulative area of SpO_2_ below the mean SpO_2_ of the recording, normalised by total duration
- SpO_2 Min_: Minimum SpO_2_ value observed during the recording

Desaturation dynamics

- DA_max sd_: Standard deviation of desaturation area, using the maximum SpO_2_ within each event as the baseline
- DL_sd_: Standard deviation of desaturation event duration
- DS_sd_: Standard deviation of desaturation slope
- DD_100 sd_: Standard deviation of desaturation depth relative to a 100% SpO_2_ baseline

Distributional characteristics of SpO_2_

- SpO_2 SK_: Skewness of the SpO_2_ signal distribution
- SpO_2 K_: Kurtosis of the SpO_2_ signal distribution

Temporal and spectral features

- PSD _peak frequency_: Frequency of maximum power within the 0.014–0.033 Hz band of the power spectral density
- PSD _band kurtosis_: Kurtosis of the power spectral density within the 0.014–0.033 Hz band
- PRSAsa: Post-anchor slope derived from phase-rectified signal averaging (PRSA)
- F2S: Relative difference between mean SpO_2_ in the first and second halves of the recording

Heart rate dynamics

- HR_ZC_: Number of zero-crossings in the heart rate signal relative to its mean value

Clinical and contextual variables

- LLS _headache_: Headache component score from the Lake Louise Score
- HAH history: Self-reported history of high-altitude headache

Feature selection frequencies varied across resampling iterations (**Supplementary Materials, Supplementary Tables 11–14, Figure 1**). Although several predictors were selected consistently, others demonstrated moderate stability. Consequently, individual feature importance should be interpreted cautiously. The predictor sets should be viewed as exploratory until validated in independent cohorts.

## Discussion

Previous studies have suggested that physiological biomarkers derived from overnight pulse oximetry may contain information relevant to AMS susceptibility, but evidence supporting prospective prediction remains limited by small sample sizes and methodological heterogeneity (Goves et al., 2024; Joyce et al., 2024). The present study extends this literature by evaluating overnight pulse oximetry-derived biomarkers, both alone and in combination with clinical and contextual metadata, in a substantially larger field cohort ascending to Everest Base Camp. Consistent with earlier observations, physiological features demonstrated predictive value prior to AMS diagnosis. Importantly, predictive performance was observed using physiological biomarkers alone, without reliance on symptom-based questionnaires, distinguishing this approach from commonly used clinical tools that track AMS only after symptoms emerge.

The ability to identify individuals at risk of AMS at Namche, approximately four days prior to reaching EBC, using biomarkers alone is particularly relevant from both physiological and practical perspectives. Preventative strategies are most effective when risk can be identified early, at altitudes where ascent profiles can be modified and individuals can be monitored more closely. In the present study, prediction at Namche was achieved using only four physiological features (**Table 4**), and inclusion of metadata did not improve model performance. Together, these findings suggest that early AMS risk may potentially be identified using a small set of objective physiological measurements, without the need for symptom-based or contextual inputs at this stage. From an implementation perspective, a model based solely on overnight pulse oximetry reduces reliance on participant-reported information and simplifies deployment within wearable or mobile-health platforms. Such an approach may facilitate automated risk assessment in remote environments where detailed clinical assessment is impractical.

At higher altitudes (Dingboche-level), prediction based on biomarkers alone remained effective. The inclusion of metadata, however, improved model performance across all metrics (**Table 3**). In this dataset, feature selection retained a larger set of variables (nine features), including both physiological and contextual inputs. This suggests that as altitude exposure increases, and symptoms begin to develop, contextual factors, such as symptom severity or clinical history, provide additional information that is not fully captured in physiological biomarkers alone. Commonly considered risk factors, including ascent rate, alcohol consumption, and prophylactic acetazolamide use, were not consistently retained by the feature-selection procedure at either location. While this does not imply that such factors are clinically unimportant, it suggests that model performance in this study was driven primarily by physiological signals derived from overnight oximetry. Consistent with this, feature selection reduced a high-dimensional feature space (>140 variables) to a small subset of informative predictors, highlighting that prediction relied on a small number of physiological signals rather than broad inclusion of all available data.

Many of the selected features are related to oxygen desaturation dynamics and variability, with some contribution from heart rate-derived dynamics at higher altitude. While these findings are consistent with known physiological responses to hypoxic exposure, feature-selection frequencies varied across resampling iterations. Consequently, individual predictors should be interpreted cautiously until their reproducibility is confirmed in larger external cohorts. Despite the evaluation of more complex machine-learning approaches (10.5281/zenodo.20428956), logistic regression demonstrated the most consistent performance across datasets. This likely reflects the modest sample size and signal-to-noise characteristics typical of biomedical field studies, in which additional model complexity can increase the risk of overfitting without improving performance. The use of an interpretable model is also advantageous for potential field applications, where transparency is important.

Several limitations should be acknowledged. Recruitment occurred at 3,440 m after participants had already completed several days of ascent. Consequently, the cohort may underrepresent individuals who developed significant altitude illness earlier in the trek or elected not to continue, potentially limiting generalisability. Although only one participant was excluded due to AMS at recruitment, some degree of survivor-selection bias cannot be excluded. In addition, ascent exposure was summarised using average rate of ascent. While this captures the overall relationship between altitude gained and time spent ascending, it does not fully account for differences in individual acclimatisation histories, such as prolonged stays at specific locations or variation in the timing of altitude exposure. Future studies may benefit from incorporating dedicated temporal variables, such as time spent at altitude or days since trek commencement, to more comprehensively characterise acclimatisation.

The dataset is modest by machine learning standards, reflecting the challenges of high-altitude field data collection. This uncertainty was reflected in the bootstrap confidence intervals around performance estimates, which remained relatively wide despite repeated cross-validation. The relatively small number of AMS events relative to the initial candidate feature set may contribute to uncertainty in feature selection and performance estimation, despite the use of nested feature selection and repeated cross-validation. External validation in independent cohorts is therefore required. Precision did not exceed 0.43 in the primary analyses, indicating that a substantial proportion of individuals identified as high risk would not subsequently develop AMS, thus supporting the use of the model as a screening or risk-stratification tool rather than a diagnostic instrument.

The cohort was predominantly composed of Fitzpatrick skin types II–IV, with limited representation of types I, V and VI (**Supplementary Material, Supplementary Table 20**), further limiting generalisability across the full spectrum of skin tones. This is particularly relevant given known limitations of pulse oximetry in individuals with darker skin pigmentation, which may influence both physiological measurements and downstream model performance (Sjoding et al., 2020; Fawzy et al., 2022; Al-Halawani et al., 2023). Future work should prioritise validation in larger, more diverse populations.

These constraints, however, should be considered within the context of the study and setting. The cohort represents a comparatively large sample for high-altitude field research and includes repeated measurements across multiple points along the ascent, enabling assessment of physiological responses under changing environmental conditions. Moreover, the study benefits from continuous overnight pulse oximetry recordings, rather than the brief spot measurements (often limited to a few minutes) commonly reported in prior studies (Kidd et al., 2013). This extended monitoring provides a more comprehensive characterisation of nocturnal oxygenation patterns.

Finally, these findings suggest several potential practical implications. Given its modest but consistent discriminative performance (**Figure 5**; **Table 3**), the model may be most appropriately positioned as a screening or risk-stratification tool rather than a standalone diagnostic instrument. Early prediction at lower altitude may support preventative strategies, such as modifying ascent profiles or increasing monitoring, while the addition of clinical information at higher altitude may further aid decision-making. The use of a simple, interpretable model with low data requirements supports potential integration into wearable or mobile-health platforms. However, this approach is intended to complement, rather than replace, existing clinical assessment and best-practice acclimatisation strategies, which remain the primary means of reducing AMS risk.

## Conclusion

Overnight pulse oximetry-derived physiological features demonstrate potential to prospectively identify individuals at risk of AMS during ascent to high altitude. Predictive signal was detectable prior to symptom onset, supporting the feasibility of objective, pre-symptomatic risk stratification. Integration of physiological monitoring with contextual clinical data may enhance decision-making at higher altitude. This approach has potential utility as a field-deployable screening tool to support early intervention strategies.

## Data Availability

The datasets presented in this study can be found in online repositories. The names of the repository/repositories and accession number(s) can be found in the article/**Supplementary Material**.
