# Supplementary material for: Prediction of acute mountain sickness from overnight pulse oximetry during ascent to Everest Base Camp: a field-based machine learning study
Source: Front Physiol. 2026 Sep 30;17:1939874. doi: 10.3389/fphys.2026.1939874 (PMC13681377; doi:10.3389/fphys.2026.1939874)
Supplement: Supplementary file 1 [file Table1.docx]

# Supplementary Materials

## 1. **Feature Definitions**

Oximetry features have been extracted from the SpO_2_ and HR signals, which have been collated from features presented by (Levy et al., 2021) and (Levy et al. 2023 and 2021), (Alvarez et al., 2010) and (Terrill, 2019). Also included are non-oximetry features. Abbreviations and units of measure are all presented for features where appropriate.

All features presented in Tables 1–9 were extracted from overnight pulse oximetry recordings using a minimally modified version of the PhysioZoo toolbox (Behar et al., 2023). The implementation followed the original algorithms for oximetry-derived feature extraction, with only minor adaptations to accommodate the study data format and preprocessing pipeline.

Features presented in Table 10 were collected during the expedition via questionnaires and medical assessments.

**Table 1. Time-Domain SpO₂ Statistics**

| **Feature** | **Definition** | **Unit** | **Notes / Source** |
| --- | --- | --- | --- |
| SpO₂ _AV_ | Mean SpO₂ (arithmetic average) | % | (Alvarez et al., 2010) |
| SpO₂ _MED_ | Median SpO₂ | % | (Alvarez et al., 2010) |
| SpO₂ _VAR_ | Variance of SpO₂ | %² | (Alvarez et al., 2010) |
| SpO₂ _SD_ | Standard deviation of SpO₂ | % | — |
| SpO₂ _CV_ | Coefficient of variation (SD / mean) | — | (Tellez et al., 2016) |
| SpO₂ _RG_ | Range of SpO₂ (max − min) | % | — |
| SpO₂ _Min_ | Minimum SpO₂ | % | — |
| SpO₂ _P_ | Percentile (default: 1st percentile) | % | (Deviaene et al., 2019) |
| SpO₂ _MAD_ | Mean absolute deviation | % | (added; standard statistical measure) |
| SpO₂ _SK_ | Skewness of SpO₂ distribution |  | (Alvarez et al., 2010) |
| SpO₂ _K_ | Kurtosis of SpO₂ distribution |  | (Alvarez et al., 2010) |

**Table 2. Signal Dynamics / Derived Indices**

| **Feature** | **Definition** | **Unit** | **Notes** |
| --- | --- | --- | --- |
| M | % of signal ≥ x% below median SpO₂ (default x=2) | % | (Deviaene et al., 2019) |
| ZC | Zero-crossing count at threshold x | count | (Xie and Minn, 2012) |
| DI | Delta index (mean absolute diff of 12s segments) | % | (Pépin et al., 1991) |
| F2S | Relative change between first and second halves of recording | % | (Tannheimer et al., 2017) |

**Table 3. Desaturation Event Features**

| **Feature** | **Definition** | **Unit** | **Notes** |
| --- | --- | --- | --- |
| No. of Desat Events | Number of desaturation events (≥4% drop, 10–60 s) | count | (Cross et al., 2015) |
| DL_u_ | Mean desaturation duration | Sec |  |
| DL_sd_ | SD of desaturation duration | sec² |  |
| ODI | Oxygen desaturation index (events per hour) | events/hr |  |
| DD_max u_ | Mean desaturation depth (event baseline) | % | (Kulkas et al., 2017) |
| DD_max sd_ | SD of desaturation depth | %² |  |
| DD_100 u_ | Mean desaturation depth (100% baseline) | % | (Terrill, 2019) |
| DD_100 sd_ | SD of desaturation depth (100% baseline) | %² |  |
| DS_u_ | Mean desaturation slope | %/s | (Levy et al., 2021) |
| DS_sd_ | SD of desaturation slope | %/s² |  |
| DA_max u_ | Mean desaturation area (event baseline) | %·s | (Kulkas et al., 2013) |
| DA_max sd_ | SD of desaturation area | %·s² |  |
| DA_100 u_ | Mean desaturation area (100% baseline) | %·s | (clarified from implementation) |
| DA_100 sd_ | SD of desaturation area (100% baseline) | %·s² |  |
| TD_u_ | Mean time between desaturations | Sec |  |
| TD_sd_ | SD of time between events | sec² |  |

**Table 4. Hypoxic Burden / Cumulative Measures**

| **Feature** | **Definition** | **Unit** | **Notes** |
| --- | --- | --- | --- |
| POD | % time spent in desaturation | % | (Kulkas et al., 2013) |
| AOD_max_ | Area under desaturation curve (event baseline, normalised) | % |  |
| AOD_100_ | Area under desaturation curve (100% baseline) | % |  |
| CA | Integral of SpO₂ below mean (AV), normalised | % |  |
| CT_50_ – CT_100_ | Cumulative time below threshold (50–100%), normalised | % | (Cross et al., 2015) |

**Table 5. Nonlinear / Complexity Metrics**

| **Feature** | **Definition** | **Unit** | **Notes** |
| --- | --- | --- | --- |
| ApEn | Approximate entropy |  | (Pincus and Singer, 1996) |
| SampEn | Sample entropy |  | (Richman and Moorman, 2000) |
| LZ | Lempel–Ziv complexity |  | (Lempel and Ziv, 1976) |
| CTM | Central tendency measure |  | (Cohen et al., 1996) (Alvarez et al., 2007) |

**Table 6. PRSA (Phase-Rectified Signal Averaging)**

| **Feature** | **Definition** | **Unit** |
| --- | --- | --- |
| PRSAc | PRSA capacity | % |
| PRSAad | PRSA amplitude difference | % |
| PRSAos | PRSA overall slope | %/s |
| PRSAsb | PRSA pre-anchor slope | %/s |
| PRSAsa | PRSA post-anchor slope | %/s |

(Source: (Bauer et al., 2006, Deviaene et al., 2019))

**Table 7. Frequency-Domain Features (SpO₂)**

**Total Spectrum**

| **Feature** | **Definition** | **Unit** |
| --- | --- | --- |
| PSD_total power_ | Total power (integral of PSD) | dB |
| PSD_total mean_ | Mean power | dB |
| PSD_total var_ | Variance of power | dB² |
| PSD_total skewness_ | Skewness of PSD distribution |  |
| PSD_total kurtosis_ | Kurtosis of PSD distribution |  |

**Table 8. Frequency-Domain Features (SpO₂)**

**Total Spectrum**

| **Feature** | **Definition** | **Unit** |
| --- | --- | --- |
| PSD_band power_ | Power within band | dB |
| PSD_relative power_ | Band power / total power |  |
| PSD_peak amplitude_ | Peak amplitude in band | dB/Hz |
| PSD_peak frequency_ | Frequency at maximum power | Hz |
| PSD_band mean_ | Mean power in band | dB |
| PSD_band variance_ | Variance in band | dB² |
| PSD_band skewness_ | Skewness of PSD in band |  |
| PSD_band kurtosis_ | Kurtosis of PSD in band |  |

**Table 9. Heart Rate (HR) Features**

(All computed analogously to SpO₂)

| **Feature** | **Definition** | **Unit** |
| --- | --- | --- |
| HR_AV_ | Mean HR | bpm |
| HR_MED_ | Median HR | bpm |
| HR_VAR_ | Variance | bpm² |
| HR_SD_ | Standard deviation | bpm |
| HR_CV_ | Coefficient of variation |  |
| HR_RG_ | Range | bpm |
| HR_Min_ | Minimum HR | bpm |
| HR_P_ | Percentile HR | bpm |
| HR_MAD_ | Mean absolute deviation | bpm |
| HR_SK_ | Skewness |  |
| HR_K_ | Kurtosis |  |
| HR_M_ | % below median threshold | % |
| HR_ZC_ | Zero crossings | count |
| HR_DI_ | Delta index | bpm |
| HR_F2S_ | First vs second half change | % |

**Table 10. Non-Oximetry Features**

| **Feature** | **Definition** | **Unit** | **Category** |
| --- | --- | --- | --- |
| Age | Age of participant at time of recording | years | Demographic |
| Sex | Biological sex of participant | Male / Female | Demographic |
| Ascent rate | Average ascent rate up to time of recording | m/day | Ascent Rate Questionnaire |
| Acclimatisation Walk | Whether participant completed an acclimatisation walk on the day prior to oximetry recording | 0/1 | Ascent Rate Questionnaire |
| Acetazolamide use | Use of acetazolamide at time of recording | 0/1 | Medication |
| Other medication use | Use of non-acetazolamide medications | 0/1 | Medication |
| Prescribed medication use | Use of prescribed medications (excluding acetazolamide) | 0/1 | Medication |
| Alcohol or drug use | Alcohol or recreational drug use within 24 hours prior to recording | 0/1 | Medication |
| AMS history | Prior history of acute mountain sickness | 0/1 | Clinical history |
| HAH history | Prior history of high-altitude headache | 0/1 | Clinical history |
| HAPE history | Prior history of high-altitude pulmonary oedema | 0/1 | Clinical history |
| HACE history | Prior history of high-altitude cerebral oedema | 0/1 | Clinical history |
| GSQS score | Groningen Sleep Quality Scale score | score | GSQS Questionnaire |
| LLS headache score | Headache component of the Lake Louise Score | score | LLSQ Questionnaire |
| AMS (outcome) | Presence of acute mountain sickness at time of medical assessment | 0/1 | Outcome |

# 2. **Feature Selection Procedures**

RFECV was used to assess feature importance and selection stability across datasets (Methods: Machine Learning Techniques). Selection frequency varied by location (Tables 10–14), with clear differences in the number and type of features retained.

In Namche, feature selection was highly consistent, with both the biomarker-only and biomarker + metadata models selecting the same four features: DA_max sd_, CT_91_, F2S, SpO₂ _Min_. These variables also showed the highest selection frequencies, indicating a stable and dominant contribution to model performance. The inclusion of metadata did not influence feature selection or alter the ranking of variables, and no additional features were introduced into the final model. This suggests that predictive performance in Namche was driven entirely by a small, robust set of physiological biomarkers, with metadata providing no incremental value.

In contrast, Dingboche exhibited a broader distribution of frequently selected features. Variables such as SpO₂ _SK_, DS_sd_, HR_ZC_, and several PSD-derived metrics were consistently ranked highly. With the inclusion of metadata, HAH history and LLSQ_Headache_ were also frequently selected, indicating a greater contribution of clinical variables at this location. The optimal models for the Dingboche-level were larger, with nine features retained in both biomarker-only and combined models. In this case, metadata contributed alongside physiological features without reducing model size, suggesting that they provided complementary rather than substitutive information.

These differences indicate that model complexity varied by dataset. In Namche, a compact feature set was sufficient to achieve strong predictive performance, suggesting that the signal was dominated by a small number of variables. At Dingboche-level, a larger number of features was required, consistent with greater variability in the data and a more distributed predictive signal.

The use of RFECV was important given the high dimensionality of the feature space (140 features within combined biomarkers + metadata datasets), enabling reduction of redundancy while limiting overfitting and identifying stable feature subsets. Across datasets, several of the most consistently selected features were derived from oxygen saturation patterns and heart rate dynamics.

Overall, these findings support the feasibility of accurate prediction using relatively small feature sets, particularly at lower altitude. The ability to achieve strong performance with a limited number of variables suggests that practical implementation may not require extensive data collection, which is advantageous in field settings.

**Table 11.** Feature ranking for the Namche biomarker-only dataset based on selection frequency during recursive feature elimination with cross-validation (RFECV). Values represent the number of cross-validation iterations in which each feature was selected.

| **Rank** | **Features** | **Selection Frequency (%)** |
| --- | --- | --- |
| 1 | DA_max sd_ | 72 |
| 2 | CT_91_ | 66 |
| 3 | F2S | 58 |
| 4 | SpO₂ _Min_ | 40 |
| 5 | CT_62_ | 30 |
| 6 | AOD_max_ | 26 |
| 7 | DD_max sd_ | 24 |
| 8 | SpO₂ _ZC_ | 22 |
| 9 | CT_86_ | 22 |
| 10 | PSD_peak frequency_ | 20 |

**Table 12.** Feature ranking for the Namche biomarker + metadata dataset based on selection frequency during recursive feature elimination with cross-validation (RFECV). Values represent the number of cross-validation iterations in which each feature was selected.

| **Rank** | **Features** | **Selection Frequency (%)** |
| --- | --- | --- |
| 1 | CT_91_ | 72 |
| 2 | DA_max sd_ | 56 |
| 3 | F2S | 48 |
| 4 | SpO₂ _Min_ | 38 |
| 5 | SpO₂ _ZC_ | 30 |
| 6 | AMS History | 28 |
| 7 | CT_62_ | 26 |
| 8 | HR_ZC_ | 24 |
| 9 | PSD_band skewness_ | 20 |
| 10 | AOD_max_ | 20 |

**Table 13.** Feature ranking for the Dingboche-level biomarker-only dataset based on selection frequency during recursive feature elimination with cross-validation (RFECV). Values represent the number of cross-validation iterations in which each feature was selected. Dingboche-level includes participants from Dingboche, Pheriche and Chhukung.

| **Rank** | **Features** | **Selection Frequency (%)** |
| --- | --- | --- |
| 1 | SpO₂ _SK_ | 74 |
| 2 | DS_sd_ | 66 |
| 3 | HR_ZC_ | 60 |
| 4 | PSD_peak frequency_ | 58 |
| 5 | PSD_band kurtosis_ | 58 |
| 6 | CA | 58 |
| 7 | DL_sd_ | 56 |
| 8 | F2S | 54 |
| 9 | DD_100 sd_ | 54 |
| 10 | HR_Med_ | 50 |

**Table 14.** Feature ranking for the Dingboche-level biomarker + metadata dataset based on selection frequency during recursive feature elimination with cross-validation (RFECV). Values represent the number of cross-validation iterations in which each feature was selected.

Dingboche-level includes participants from Dingboche, Pheriche and Chhukung.

| **Rank** | **Features** | **Selection Frequency (%)** |
| --- | --- | --- |
| 1 | SpO₂ _SK_ | 84 |
| 2 | PSD_band kurtosis_ | 82 |
| 3 | DL_sd_ | 74 |
| 4 | SpO₂ _K_ | 74 |
| 5 | HAH History | 70 |
| 6 | PRSA_sa_ | 70 |
| 7 | F2S | 70 |
| 8 | HR_ZC_ | 68 |
| 9 | Headache Score | 64 |
| 10 | SpO₂ _M_ | 58 |

**Figure 1.** Frequency plots for logistic regression models in Namche and Dingboche with selected input feature set. Shaded bars show features shared across dataset per location. Dingboche referenced in the figure refers to the “Dingboche-level” which included participants from Dignboche, Pheriche and Chhukung.

**3. Sensitivity analysis for LLS Headache Score**

**Table 15.** Cross‑validated predictive performance of logistic regression models for acute mountain sickness (AMS) prediction at Dingboche-level. Models were trained using overnight pulse oximetry-derived physiological biomarkers alone, or in combination with demographic and clinical metadata. To assess the potential influence of definitional overlap between LLS headache and the AMS outcome, model performance was compared before and after exclusion of the LLS headache variable. Values represent mean area under the receiver operating characteristic curve (AUC) obtained from repeated stratified cross-validation.

| **Model** | **Features** | **AUC (mean ± SD)s** |
| --- | --- | --- |
| Biomarker + Metadata (including headache) | PSD*_band kurtosis_*, DL*_sd_*, HAH History, PRSAsa, F2S, HR*_ZC_*, LLS*_Headache_*, SpO₂ *_SK_*, SpO₂ *_K_* | 0.84 ± 0.08 (95% CI 0.76-0.91) |
| Biomarker + Metadata (excluding headache) | PSD*_band kurtosis_*, DL*_sd_*, HAH History, PRSAsa, F2S, HR*_ZC_*, SpO₂ *_SK_*, SpO₂ *_K_* | 0.81 ± 0.09  (95% CI 0.72-0.90) |
| Biomarker Only | DS*_sd_*, HR*_ZC_* , PSD*_peak frequency_*, PSD*_band kurtosis_*, CA, DL*_sd_*, F2S, DD*_100_sd_*, SpO*2 _SK_* | 0.74 ± 0.10 (95% CI 0.63-0.84) |

**4. Model selection expanded results.**

**Table 16.** Comparison of classifier performance for the final Namche biomarker only models.

| **Classifier** | **Validation AUC (mean ± SD)** | **Training AUC (mean ± SD)** |
| --- | --- | --- |
| Logistic Regression | 0.74 ± 0.08 | 0.78 ± 0.02 |
| Linear SVM | 0.73 ± 0.09 | 0.78 ± 0.02 |
| Random Forest | 0.69 ± 0.12 | 1.00 ± 0.00 |
| Decision Tree | 0.57 ± 0.09 | 1.00 ± 0.00 |
| Gradient Booster | 0.64 ± 0.11 | 1.00 ± 0.00 |

**Table 17.** Comparison of classifier performance for the final Namche biomarker + metadata models.

| **Classifier** | **Validation AUC (mean ± SD)** | **Training AUC (mean ± SD)** |
| --- | --- | --- |
| Logistic Regression | 0.74 ± 0.08 | 0.78 ± 0.02 |
| Linear SVM | 0.73 ± 0.09 | 0.78 ± 0.02 |
| Random Forest | 0.69 ± 0.11 | 1.00 ± 0.00 |
| Decision Tree | 0.57 ± 0.09 | 1.00 ± 0.00 |
| Gradient Booster | 0.64 ± 0.10 | 1.00 ± 0.00 |

**Table 18.** Comparison of classifier performance for the final Dingboche-level biomarker only models.

| **Classifier** | **Validation AUC (mean ± SD)** | **Training AUC (mean ± SD)** |
| --- | --- | --- |
| Logistic Regression | 0.74 ± 0.11 | 0.84 ± 0.02 |
| Linear SVM | 0.69 ± 0.12 | 0.79 ± 0.03 |
| Random Forest | 0.75 ± 0.10 | 1.00 ± 0.00 |
| Decision Tree | 0.63 ± 0.09 | 1.00 ± 0.00 |
| Gradient Booster | 0.70 ± 0.08 | 1.00 ± 0.00 |

**Table 19.** Comparison of classifier performance for the final Dingboche-level biomarker + metadata models.

| **Classifier** | **Validation AUC (mean ± SD)** | **Training AUC (mean ± SD)** |
| --- | --- | --- |
| Logistic Regression | 0.84 ± 0.08 | 0.92 ± 0.02 |
| Linear SVM | 0.80 ± 0.09 | 0.86 ± 0.02 |
| Random Forest | 0.75 ± 0.12 | 1.00 ± 0.00 |
| Decision Tree | 0.61 ± 0.11 | 1.00 ± 0.00 |
| Gradient Booster | 0.76 ± 0.11 | 1.00 ± 0.00 |

**5. Performance diagnostics for the final logistic regression models.**

**Figure 2.** Performance diagnostics for the final logistic regression models. (A-B) Distribution of AUC and accuracy across repeated stratified 5-fold cross-validation (10 repeats × 5 folds). Boxes represent the interquartile range, centre lines indicate the median, whiskers indicate the non-outlier range, and points represent individual validation folds. (C) Receiver operating characteristic (ROC) curves and (D) precision-recall curves derived from pooled out-of-fold predictions.

## 6. **SHAP Analysis**

Model explainability was assessed using SHapley Additive exPlanations (SHAP). Feature contributions for the final logistic regression models were summarised using SHAP beeswarm plots (Figures 3-4). Points represent individual observations, coloured by feature value (low to high), with position on the x-axis indicating the SHAP value (change in log-odds). Positive values increase predicted AMS risk, while negative values decrease it. Features are ordered by mean absolute SHAP value within each model.

SHAP analysis was conducted on the final feature subsets identified through the feature selection procedure described in the main manuscript. As such, results reflect the relative contribution and directionality of features within each selected model, rather than importance across the full candidate feature space.

For the Namche models, as discussed in the manuscript, feature selection identified the same four variables for both the biomarker only model and the combined biomarker + metatdata models. Figure 3 shows the SHAP beeswarm plot for the final Namche model. Feature contributions showed relatively narrow distributions with limited inter-individual variability, and higher-ranked features were clearly separated from lower-ranked features.

**Figure 3. SHAP summary plots for logistic regression models in Namche with selected input feature set.** Figure shows feature contributions to model predictions for the selected feature set for Namche datasets (from biomarkers alone and combined with metadata). Points represent individual samples, coloured by feature value (low to high). Horizontal position indicates the SHAP value (change in log-odds), with positive values increasing predicted risk and negative values decreasing it. Features are ordered by mean absolute SHAP value within each model.

In contrast, Dingboche-level showed a broader distribution of feature contributions (Figure 4, panels A and B). SHAP values were spread across a larger number of selected features, with greater overlap in magnitude and wider dispersion across individuals. This suggests that predictions at higher altitude depended on a more distributed combination of inputs rather than a small number of dominant variables. When metadata were included, these variables ranked among the higher contributing predictors, with symptom-based features showing contributions comparable to several physiological variables. Oximetry-derived features continued to contribute alongside these, indicating that metadata provided complementary information rather than replacing physiological inputs.

**Figure 4. SHAP summary plots for logistic regression models at Dingboche-level across input feature sets.** Each panel shows feature contributions to model predictions for Dingboche-level datasets using biomarkers alone or combined with metadata. Points represent individual samples, coloured by feature value (low to high). Horizontal position indicates the SHAP value (change in log-odds), with positive values increasing predicted risk and negative values decreasing it. Features are ordered by mean absolute SHAP value within each model. “Dingboche” referenced in the figure refers to the “Dingboche-level” which included participants from Dingboche, Pheriche and Chhukung.

## 7. **Fitzpatrick Skin Type**

Fitzpatrick skin type distribution for the full study cohort (n = 209) is presented in Table 20 and indicates limited representation of darker skin tones. While the ML model was trained on a subset of this cohort, detailed reconstruction of the Fitzpatrick distribution within that subset was not feasible. However, no targeted sampling by skin type was performed. Given the overall narrow distribution observed in the full cohort, substantial diversity within the ML subsets is unlikely.

**Table 20.** Distribution of Fitzpatrick skin types in the full study cohort (n = 209)

| **Fitzpatrick Skin Type** | **Score Range** | **n** | **%** |
| --- | --- | --- | --- |
| **I** | 0-7 | 1 | 0.5 |
| **II** | 8-16 | 40 | 19.1 |
| **III** | 17-25 | 117 | 56.0 |
| **IV** | 26-30 | 42 | 20.1 |
| **V-VI*** | > 30 | 9 | 4.3 |

* Types V and VI were grouped due to low numbers.

## 8. **Threshold Analysis**

Classification metrics (precision, sensitivity, F1 score, and specificity) are reported across a range of decision thresholds using pooled out-of-fold predictions from repeated cross-validation. Lower thresholds increase sensitivity at the expense of specificity, consistent with a screening-oriented approach.

**Table 21.** Threshold-dependent classification performance of the Namche model (physiological biomarkers only).

| **Threshold** | **Precision** | **Sensitivity** | **F1** | **Specificity** |
| --- | --- | --- | --- | --- |
| **0.1** | 0.24 | 1 | 0.38 | 0.10 |
| **0.15** | 0.24 | 1 | 0.39 | 0.15 |
| **0.2** | 0.26 | 1 | 0.41 | 0.21 |
| **0.25** | 0.26 | 0.96 | 0.41 | 0.26 |
| **0.3** | 0.28 | 0.92 | 0.42 | 0.34 |
| **0.35** | 0.29 | 0.85 | 0.43 | 0.43 |
| **0.4** | 0.32 | 0.80 | 0.45 | 0.53 |
| **0.45** | 0.35 | 0.77 | 0.48 | 0.60 |
| **0.5** | 0.37 | 0.69 | 0.48 | 0.67 |
| **0.55** | 0.39 | 0.6 | 0.47 | 0.74 |
| **0.6** | 0.44 | 0.51 | 0.48 | 0.82 |
| **0.65** | 0.43 | 0.39 | 0.41 | 0.86 |
| **0.7** | 0.40 | 0.27 | 0.32 | 0.89 |
| **0.75** | 0.46 | 0.20 | 0.28 | 0.93 |
| **0.8** | 0.50 | 0.14 | 0.21 | 0.96 |
| **0.85** | 0.40 | 0.06 | 0.10 | 0.98 |
| **0.9** | 0.35 | 0.03 | 0.05 | 0.99 |

**Table 22.** Threshold-dependent classification performance of the Dingboche-level model using physiological biomarkers. Model performance demonstrates improved balance between sensitivity and specificity compared to the Namche model.

| **Threshold** | **Precision** | **Sensitivity** | **F1 Score** | **Specificity** |
| --- | --- | --- | --- | --- |
| **0.1** | 0.22 | 0.94 | 0.35 | 0.14 |
| **0.15** | 0.23 | 0.92 | 0.37 | 0.21 |
| **0.2** | 0.25 | 0.92 | 0.39 | 0.29 |
| **0.25** | 0.27 | 0.89 | 0.41 | 0.39 |
| **0.3** | 0.28 | 0.85 | 0.42 | 0.45 |
| **0.35** | 0.30 | 0.78 | 0.43 | 0.53 |
| **0.4** | 0.32 | 0.74 | 0.45 | 0.61 |
| **0.45** | 0.35 | 0.67 | 0.46 | 0.68 |
| **0.5** | 0.37 | 0.61 | 0.46 | 0.74 |
| **0.55** | 0.40 | 0.57 | 0.47 | 0.79 |
| **0.6** | 0.44 | 0.52 | 0.48 | 0.83 |
| **0.65** | 0.46 | 0.44 | 0.45 | 0.87 |
| **0.7** | 0.46 | 0.36 | 0.40 | 0.89 |
| **0.75** | 0.48 | 0.29 | 0.36 | 0.92 |
| **0.8** | 0.47 | 0.23 | 0.31 | 0.94 |
| **0.85** | 0.44 | 0.15 | 0.23 | 0.95 |
| **0.9** | 0.46 | 0.09 | 0.15 | 0.97 |

**Table 23.** Threshold-dependent classification performance of the Dingboche-level model combining physiological biomarkers and metadata. This model demonstrates the most favourable sensitivity–specificity trade-off, supporting its use for risk stratification at higher altitude.

| **Threshold** | **Precision** | **Sensitivity** | **F1 Score** | **Specificity** |
| --- | --- | --- | --- | --- |
| **0.1** | 0.27 | 1 | 0.43 | 0.45 |
| **0.15** | 0.29 | 0.995 | 0.45 | 0.51 |
| **0.2** | 0.32 | 0.985 | 0.48 | 0.56 |
| **0.25** | 0.33 | 0.965 | 0.49 | 0.60 |
| **0.3** | 0.36 | 0.93 | 0.52 | 0.65 |
| **0.35** | 0.38 | 0.90 | 0.54 | 0.70 |
| **0.4** | 0.39 | 0.84 | 0.53 | 0.73 |
| **0.45** | 0.40 | 0.77 | 0.52 | 0.76 |
| **0.5** | 0.40 | 0.73 | 0.52 | 0.78 |
| **0.55** | 0.41 | 0.65 | 0.50 | 0.81 |
| **0.6** | 0.42 | 0.58 | 0.48 | 0.83 |
| **0.65** | 0.41 | 0.51 | 0.45 | 0.85 |
| **0.7** | 0.41 | 0.46 | 0.43 | 0.87 |
| **0.75** | 0.39 | 0.36 | 0.37 | 0.88 |
| **0.8** | 0.40 | 0.255 | 0.31 | 0.922 |
| **0.85** | 0.42 | 0.170 | 0.24 | 0.952 |
| **0.9** | 0.41 | 0.120 | 0.19 | 0.965 |

## 9. **Calibration Analysis**

Model calibration was assessed using calibration curves, Brier scores, calibration intercepts, and calibration slopes (Table 24). Brier scores ranged from 0.157 to 0.208. Calibration intercepts were negative across all models (-1.24 to -1.30), indicating a tendency to overestimate risk, while calibration slopes ranged from 0.53 to 0.77, suggesting that predicted probabilities were somewhat too extreme. The calibration curve for the best performing model (Dingboche-level Biomarker + Metadata) is also presented in Figure 5. It demonstrates increasing overestimation at higher predicted probabilities.

**Table 24. Calibration metrics for the final logistic regression models.** Calibration was assessed using pooled out-of-fold predictions generated during repeated stratified cross-validation.

| **Model** | **Brier Score** | **Calibration Intercept** | **Calibration Slope** |
| --- | --- | --- | --- |
| **Namche** | 0.208 | -1.24 | 0.77 |
| **Dingboche-level Biomarkers** | 0.199 | -1.27 | 0.53 |
| **Dingboche-level Biomarker +Metadata** | 0.157 | -1.3 | 0.66 |

**Figure 5.** Calibration curve for the logistic regression model at Dingboche-level using biomarkers + metadata, calculated using pooled out-of-fold predictions. The model demonstrates reasonable calibration overall, with a tendency to overestimate risk at higher predicted probabilities.

## 10.  **Inter-feature Correlation Matrices**

Correlations among predictors in the Namche model were uniformly weak (maximum |r| = 0.16), while correlations in the Dingboche models were generally weak to moderate (maximum |r| = 0.58), indicating limited redundancy among retained predictors.

**Figure 6. Inter-feature correlation matrix for predictors included in the final Namche logistic regression model.** Heatmap showing pairwise Pearson correlation coefficients between predictors retained in the final model. Positive correlations are shown in red and negative correlations in blue, with colour intensity proportional to correlation magnitude. “Min” shown on the heatmap is Minimum SpO₂.

**Figure 7. Inter-feature correlation matrix for predictors included in the final Dingboche biomarker logistic regression model.** Heatmap showing pairwise Pearson correlation coefficients between predictors retained in the final model. Positive correlations are shown in red and negative correlations in blue, with colour intensity proportional to correlation magnitude. SK and K are the skewness and kurtosis of SpO₂.

**Figure 8. Inter-feature correlation matrix for predictors included in the final Dingboche biomarker + metadata logistic regression model.** Heatmap showing pairwise Pearson correlation coefficients between predictors retained in the final model. Positive correlations are shown in red and negative correlations in blue, with colour intensity proportional to correlation magnitude. SK and K are the skewness and kurtosis of SpO₂.

**References**

Alvarez, D., Hornero, R., García, M., del Campo, F., and Zamarrón, C. (2007). Improving diagnostic ability of blood oxygen saturation from overnight pulse oximetry in obstructive sleep apnea detection by means of central tendency measure. *Artif Intell Med* 41(1)**,** 13-24. doi: 10.1016/j.artmed.2007.06.002.

Alvarez, D., Hornero, R., Marcos, J.V., and del Campo, F. (2010). Multivariate analysis of blood oxygen saturation recordings in obstructive sleep apnea diagnosis. *IEEE Trans Biomed Eng* 57(12)**,** 2816-2824. doi: 10.1109/tbme.2010.2056924.

Behar, J., Levy, J., Zvuloni, E., Gendelman, S., Rosenberg, A., Biton, S., et al. (2023). PhysioZoo: The Open Digital Physiological Biomarkers Resource. *Quantitative Biology*. doi: <https://doi.org/10.48550/arXiv.2309.04498>.

Cohen, M.E., Hudson, D.L., and Deedwania, P.C. (1996). Applying continuous chaotic modeling to cardiac signal analysis. *IEEE Engineering in Medicine and Biology Magazine* 15(5)**,** 97-102. doi: 10.1109/51.537065.

Cross, T.J., Ross-Keller, M., Issa, A., Wentz, R., Taylor, B., and Johnson, B.D. (2015). The Impact of Averaging Window Length on the “Desaturation” Indexes Obtained Via Overnight Pulse Oximetry at High Altitude. *SLEEP* 38(8)**,** 1331-1334.

Deviaene, M., Testelmans, D., Buyse, B., Borzee, P., Van Huffel, S., and Varon, C. (2019). Automatic Screening of Sleep Apnea Patients Based on the SpO(2) Signal. *IEEE J Biomed Health Inform* 23(2)**,** 607-617. doi: 10.1109/jbhi.2018.2817368.

Kulkas, A., Duce, B., Leppanen, T., Hukins, C., and Toyras, J. (2017). Severity of desaturation events differs between hypopnea and obstructive apnea events and is modulated by their duration in obstructive sleep apnea. *Sleep Breath* 21(4)**,** 829-835. doi: 10.1007/s11325-017-1513-6.

Kulkas, A., Tiihonen, P., Julkunen, P., Mervaala, E., and Töyräs, J. (2013). Novel parameters indicate significant differences in severity of obstructive sleep apnea with patients having similar apnea-hypopnea index. *Med Biol Eng Comput* 51(6)**,** 697-708. doi: 10.1007/s11517-013-1039-4.

Lempel, A., and Ziv, J. (1976). On the Complexity of Finite Sequences. *IEEE Transactions on Information Theory* 22(1)**,** 75-81. doi: 10.1109/TIT.1976.1055501.

Levy, J., Álvarez, D., Rosenberg, A.A., Alexandrovich, A., Del Campo, F., and Behar, J.A. (2021). Digital oximetry biomarkers for assessing respiratory function: standards of measurement, physiological interpretation, and clinical use. *NPJ Digit Med* 4(1)**,** 1. doi: 10.1038/s41746-020-00373-5.

Pépin, J.L., Lévy, P., Lepaulle, B., Brambilla, C., and Guilleminault, C. (1991). Does Oximetry Contribute to the Detection of Apneic Events?: Mathematical Processing of the SaO<sub>2</sub> Signal. *CHEST* 99(5)**,** 1151-1157. doi: 10.1378/chest.99.5.1151.

Pincus, S., and Singer, B.H. (1996). Randomness and degrees of irregularity. *Proc Natl Acad Sci U S A* 93(5)**,** 2083-2088. doi: 10.1073/pnas.93.5.2083.

Richman, J.S., and Moorman, J.R. (2000). Physiological time-series analysis using approximate entropy and sample entropy. *American Journal of Physiology-Heart and Circulatory Physiology* 278(6)**,** H2039-H2049. doi: 10.1152/ajpheart.2000.278.6.H2039.

Tannheimer, M., van der Spek, R., Brenner, F., Lechner, R., Steinacker, J.M., and Treff, G. (2017). Oxygen saturation increases over the course of the night in mountaineers at high altitude (3050–6354 m). *Journal of Travel Medicine* 24(5)**,** 1-6.

Tellez, H.F., Morrison, S.A., Neyt, X., Mairesse, O., Piacentini, M.F., Macdonald-Nethercott, E., et al. (2016). Exercise during Short-Term and Long-Term Continuous Exposure to Hypoxia Exacerbates Sleep-Related Periodic Breathing. *Sleep* 39(4)**,** 773-783. doi: 10.5665/sleep.5626.

Terrill, P.I. (2019). A review of approaches for analysing obstructive sleep apnoea-related patterns in pulse oximetry data. *Respirology*. doi: 10.1111/resp.13635.

Xie, B., and Minn, H. (2012). Real-time sleep apnea detection by classifier combination. *IEEE Trans Inf Technol Biomed* 16(3)**,** 469-477. doi: 10.1109/titb.2012.2188299.
